# Head and face anthropometric study for respirators in the multi-ethnic Asian population of Malaysia

**DOI:** 10.3389/fpubh.2022.972249

**Published:** 2022-08-26

**Authors:** Yin Cheng Lim, Ameerah Su'ad Abdul Shakor, Nadia Mohamad, Muhammad Alfatih Pahrol, Rohaida Ismail, Zhuo Lin Chong, Mohd Hatta Abdul Mutalip, Mohd Azahadi Omar, Mahmoud Danaee, Guo Tung Wan, Rafiza Shaharudin

**Affiliations:** ^1^Environmental Health Research Centre, Institute for Medical Research, National Institutes of Health, Ministry of Health, Selangor, Malaysia; ^2^Department of Social and Preventive Medicine, Faculty of Medicine, University of Malaya, Kuala Lumpur, Malaysia; ^3^Institute for Public Health, National Institutes of Health, Ministry of Health, Selangor, Malaysia; ^4^Sector for Biostatistics and Data Repository, National Institutes of Health, Ministry of Health, Selangor, Malaysia; ^5^Department of Pharmacy, Sungai Buloh Hospital, Selangor, Malaysia

**Keywords:** bivariate, face dimensions, facial size, respiratory fits test, respirators sizing, craniofacial

## Abstract

**Background:**

Existing anthropometric studies for respirator designs are based on the head and facial dimensions of Americans and Chinese nationals, with no studies for multi-ethnic countries like Malaysia. This study aimed to create head and facial morphological database for Malaysia, specifically to identify morphological differences between genders, ethnicities, and birthplaces, as well as predictors of the dimensions.

**Design:**

A cross-sectional study.

**Setting:**

Malaysia.

**Participants:**

A nation-wide cross-sectional study using a complex survey design with two stage-stratified random sampling was conducted among 3,324 participants, aged 18 years and above who were also participants of the National Health and Morbidity Survey 2020.

**Primary and secondary outcomes:**

The study collected data on sociodemographic, measurement of Body Mass Index (BMI) and 10 head and facial dimensions (3 dimensions were measured using direct measurement, and 7 others using Digimizer software for 2-dimension images). Linear regression was performed to determine the association between gender, ethnicity, birthplace, age and BMI and the dimensions.

**Results:**

There were significant differences in all the dimensions between sex, birthplace and ethnicity (*p* < 0.005). Further analysis using linear regression showed sex, ethnicity, birthplace, age and BMI were significant predictors of the dimensions. In comparison to studies from the United States and China, our study population had a wider interpupillary distance and nose breadth for both male and female participants, but smaller bigonial breadth and smaller minimal frontal breadth.

**Conclusion:**

These findings could assist in the design and sizing of respirators that will fit Malaysians and possibly other Southeast Asian population.

## Background

The respirator is an essential element of human respiratory Personal Protective Equipment (PPE) against airborne pollutants. However, in order to be an efficient PPE, respirators must appropriately suit the size and shape of users' faces; otherwise, pollutants will enter the users' respiratory system and endanger their lives. As a result, ensuring an appropriate fit of the respirator for the target population is a crucial challenge in developing respirators.

A correctly fitting respirator is determined by several factors, including user's head and facial size, design of the respirator, and user's activities while wearing the respirator. Among these, facial dimensions in relation to respirator design have been discovered to be critical in inward leakage of respirators (1, 2). Head and facial dimensions have been shown to differ depending on ethnicity, gender, age, BMI, and geographic location (3–6). The study among United State of America's population indicated significant differences in head and facial measurements across ethnic groups and sex (3). A study among lecturers and students at a University in China showed that Chinese have shorter and wider facial dimension than Americans (4). Even within China, the studies also showed there were differences in the measurements of bitragion chin arc, face length, nose length, and nose protrusion between workers born in the north as compared to workers born in the south (5). Another study compared facial dimensions between Koreans and Japanese and found that the later had greater minimal frontal breadth, interpupillary distance, face length, nose breath, and nose height (6). Differences in head and facial dimensions were observed as the population aged, with participants aged 45 years and older having greater size in 13 facial dimensions as compared to those aged between 18 and 29 years old, with the most prominent difference being in the lip length, nose breadth, and subnasale-sellion length (3).

To ensure proper fit of respirators to targeted or intended users, new models of respirators in the United States and China are required to meet the requirements of certification from National Institute Occupational Safety and Health (NIOSH) and the State Administration of Work Safety, respectively, prior to marketing (7). The facepiece of respirators is fit-tested on a panel of human subjects with head and facial sizes representative of the user population, conventionally being the population in the nation of the geographical area where the respirators are designed and manufactured. The respirator fit test panel (RFTP) is typically used as a matrix for selection of candidates to serve as the representative test subjects in this process (7). In the late 1960s, the Respirator Research and Development Section of the Los Alamos National Laboratory (LANL) developed RFTPs for fit testing based on head and facial anthropometrics of personnel serving in the United States' Air Force (8). The full facepiece fit test panel was based on the bivariate distribution of face length and face width while the half facepiece fit test panel was based on the bivariate distribution of the face length and lip length. There has long been a concern about the applicability of fit test panels, generated from military personnel, for civilian workers.

In 2003, the National Institute for Occupational Safety and Health (NIOSH) had conducted a head-and-face anthropometric survey on 3997 US civilian workers (9). Based on the results of this large-scaled anthropometric survey, a bivariate panel using face length and face width, as well as a principal component analysis (PCA) panel using 10 head and facial dimensions, were created. The study revealed that the 10 dimensions were associated with respirator fit and leakage and predicted well the remaining head and facial dimensions (9). Therefore, respirators designed to fit these panels were expected to suitably accommodate more than 95% of the current US civilian workers. The authors concluded that both panels were more representative of the US population than the existing LANL panel. The inclusion of the eight additional head and facial dimensions allows the PCA panel to provide better criteria for excluding extreme face sizes from being used. In China, two Chinese RFTPs were developed following the methods adopted in developing the NIOSH bivariate and PCA RFTPs (10). The RFTP was found to be able to accommodate at least 95% of the surveyed Chinese workers. The findings also showed that up to 35% of Chinese residents were excluded from test panels developed for an American workforce (10).

Locally, there is a lack of evidence on head and facial anthropometric data, despite several published studies among Malaysian (11–16). Generally, their study sample size was quite small with <300 participants and it did not include all ethnicities. Two of the studies focused solely on nasolabial and mentolabial dimensions (11, 16), whereas another study attempted to compare differences in the nasofacial length and width between three races in Malaysia (15). Moreover, there are no available data from the neighboring countries as well, except one study conducted among Javanese females (17). The study found that there were significant disparities in the facial dimensions between Javanese and white women.

Given that findings from previous studies showed there were differences in the head and facial dimensions of population from different continents, and no previous head and facial anthropometric studies for multi-ethnicity nations such as Malaysia have been conducted, a study aimed to establish head and facial morphological database for Malaysians was conducted. It also aimed to identify morphological differences among gender, age, ethnicity and birthplace and to determine the predictors that may influence head and facial dimensions.

## Methodology

### Study design

This was a nation-wide population based cross-sectional study using complex sample design involving direct measurement and analysis of 2D photogrammetry among participants in the National Health and Morbidity Survey (NHMS) 2020 by the Institute for Public Health, National Institute of Health Malaysia. The detailed description of sampling method had been described in NHMS 2020 report (18). Sampling frame for this study were based on the updated National Population and Housing Census 2020, provided by Department of Statistics Malaysia (DOSM). It included Malaysian residents of non-institutionalized living quarters in both rural and urban areas from all 13 states and three Federal Territories. Malaysia was divided into Enumeration Blocks (EB), which are arbitrarily geographically contiguous areas with defined boundaries. For each EB has an average of 80–120 Living Quarters (LQ) and a population of 500–600 people of all ages. A cluster size of 20 LQs were selected from each selected EB.

We employed two-stage stratified random sampling. The first stratum included all Malaysian states, and the second stratum included urban and rural localities within each state. The sampling processes was divided into two stages: the Primary Sampling Unit (PSU), which is the Enumeration Block (EBs), and the Secondary Sampling Unit (SSU), which is the Living Quarters (LQs) within the chosen EBs. The PSU and SSU were randomly selected by DOSM based on the required sample size. A total of 2,260 LQs were chosen from 113 EBs in Malaysia (83 EBs from Peninsular Malaysia, 13 EBs from Sabah, and 17 EBs from Sarawak). Participants were allocated proportionately to states, urban and rural, based on population size. Thus, more samples were allocated to states with larger population sizes, such as Selangor, Johor, Sabah, and Sarawak. A lesser number of samples were allocated to states with smaller population sizes, such as Perlis, Melaka, and Wilayah Persekutuan Putrajaya.

### Selection of participants

We used scouts to identify specific LQs from each EB. Scouting activities were based on DOSM-supplied maps and household lists. We identified selected LQs from each EB, updated the list of household members, and tagged the selected LQs. The survey was then informed to the head of household, household members, community leader and relevant government bodies. We contacted survey participants to schedule a team visit and survey appointment. Any Malaysian resident of the area who was 18 years old and older was eligible to participate. Participants with dental or facial deformities, as well as those who objected to shaving their beard and mustache, were excluded from taking part.

### Data collection

#### Questionnaire

Socio-demographic data on age, sex, ethnicity and birthplaces was collected through a self-administered questionnaire.

#### Physical examination

Physical examination was performed by trained research assistants. Weight was measured to the nearest 0.1 kg using a digital scale (TANITA model HD 309) which has the intracluster Correlation (ICC) of 0.99 (19) and height was measured using a tape measure.

#### Direct measurement procedure for three head and facial dimensions

The 10 selected morphological points were located by inspection and/or palpation in accordance with the 1988 Anthropometric Survey of the US Army Personnel Project (20). Spreading calipers were used to measure head breadth, bizygomatic breadth and bigonial breadth, which is considered the gold standard for head and facial measurement (21) (Table 1). During the measurement process, the investigators attempted to ensure that the participants were comfortable and sitting with a natural head position and relaxed lips.

**Table 1:** Head and facial dimensions and landmarks [7].

**Dimension**	**Tools**	**Description**	**Diagram**
1. Bigonial Breadth	caliper	Distance between the right and left gonion	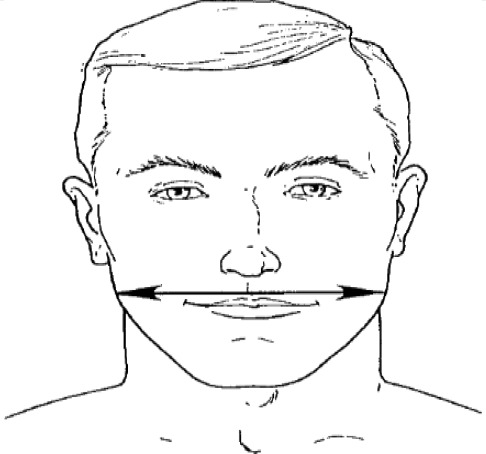
2. Bizygomatic breadth	caliper	Maximum horizontal breadth of the face	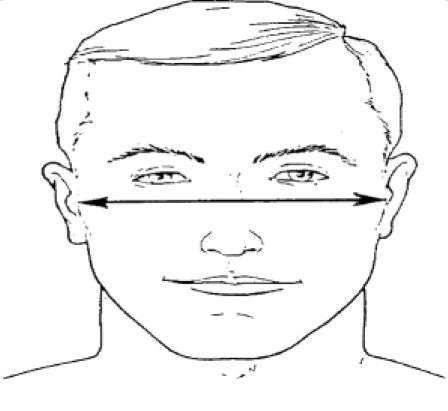
3. Head breadth	caliper	Maximum horizontal breadth of the head	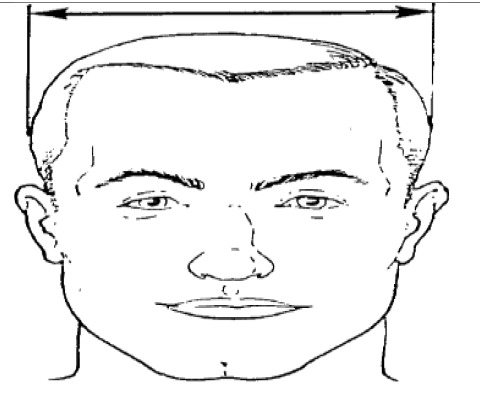
4. Interpupillary distance	2D photogrammetry	Distance between the center of pupil	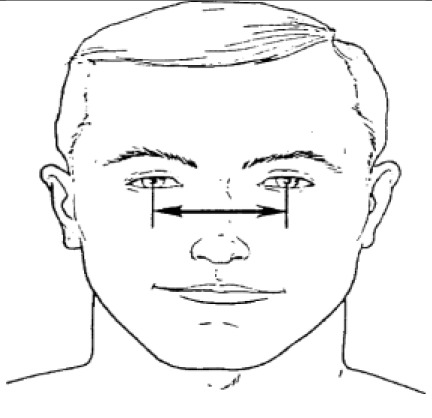
5. Menton-sellion length	2D photogrammetry	Distance between the menton and the sellion	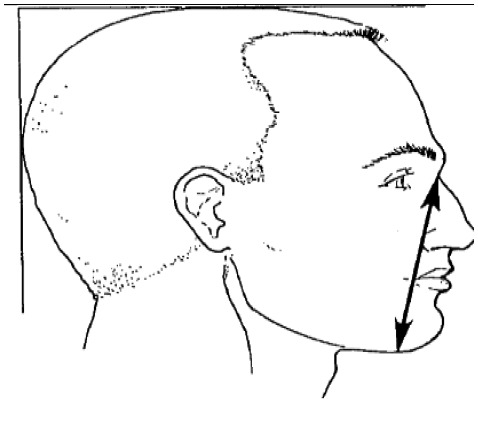
6. Minimum frontal breadth	2D photogrammetry	Distance between the right and left frontotemporal	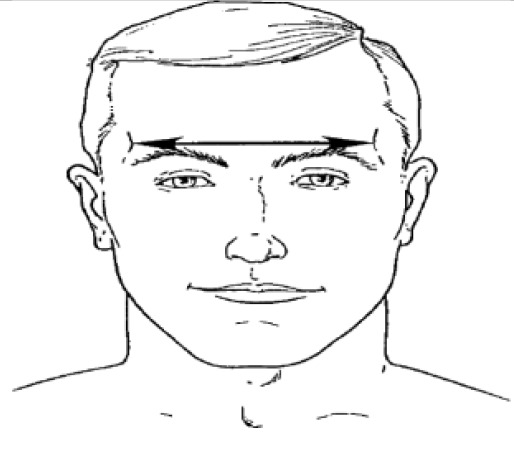
7. Nasal root breadth	2D photogrammetry	Horizontal breadth of nose at the sellion	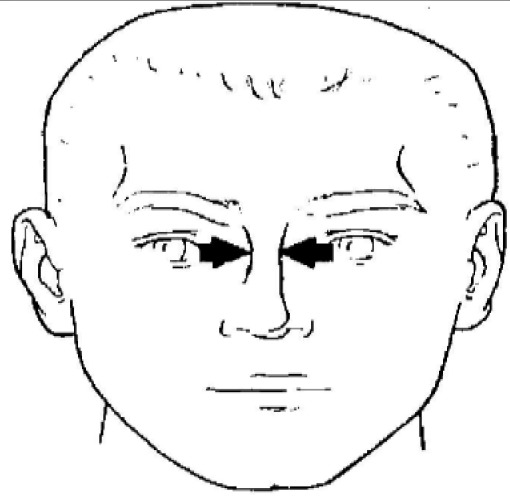
8. Nose breadth	2D photogrammetry	Distance between the right and left alare	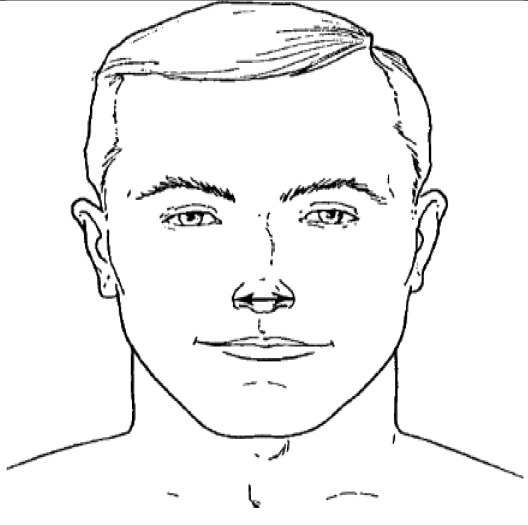
9. Nose protrusion	2D photogrammetry	Distance between the pronasale and the subnasale	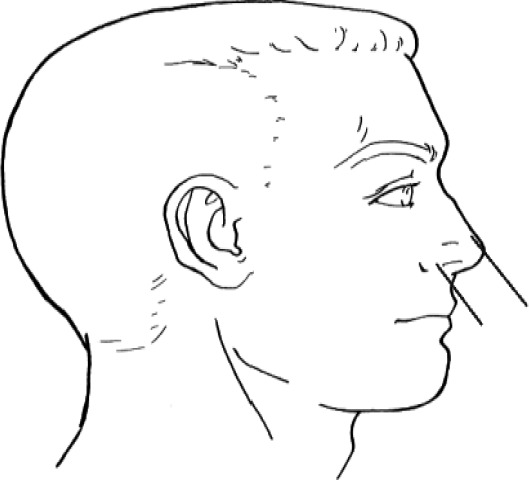
10. Subnasale- sellion length	2D photogrammetry	Distance between the subnasale and the sellion	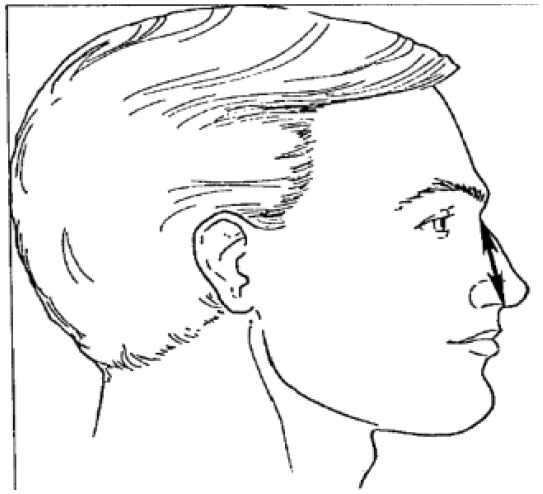

#### 2D photogrammetry procedure for seven head and facial dimensions

The participants' images were captured using a 20.0-megapixel digital camera (Canon IXUS 190, Tokyo, Japan) positioned on a tripod (Manfrotto MKCOMPACTLT-BK, Cassola, Italy) at a fixed distance of 1.0 m. For each participant, one anterior and one lateral photo were taken.

All images were captured in JPEG format and were transferred to a computer after each day of shooting. The anthropometric dimensions were calculated using the software package Digimizer version 5.4.4. Details of 2D photogrammetry methods can be found in previously published paper (22). This method has been found to have high reliability (ICC ranged between 0.85 and 0.99), and high validity as demonstrated by the Bland-Altman analyses for the seven head and facial dimensions.

### Statistical analysis

Complex sample analysis was used to obtain prevalence and population estimates with 95% confidence intervals. Prior to the analysis, sample weights were calculated for each respondent to improve the sample's representativeness in terms of the size, distribution, and characteristics of the study population. Basic weight for each sampled household would be the inverse of its selection probability (calculated by multiplying the probabilities at each sampling stage). To calculate the sample weights, the basic weight was adjusted based on the non-response and post-stratification factors.

Statistical Package for Social Science SPSS Statistics (SPSS) version 26 was used for analysis. Descriptive analysis for continuous data was presented as mean with standard deviation considering the dataset was large and normality was assumed. Categorical data were presented in frequency and column percentage. The independent sample t-test was used to calculate the difference in means between groups whereas Cohen's d was used to determine effect size due to the large sample size.

Linear regression analysis was conducted to determine the effect of age, gender, ethnicity, birthplace and BMI on head and facial dimensions. Ten head and facial dimensions served as dependent variables, while sex, ethnicity, birthplace, age, and BMI served as independent variables. Age and BMI were treated as continuous variables. Sex, ethnicity, and birthplaces were treated as categorical data with two categories. Female, non-Malay and birthplace in West Malaysia were treated as the reference group.

Cluster analysis was employed to optimize and group participants based on multivariate similarities across samples. The Multivariate Analysis of Variance (MANOVA) test was used to determine the number of groups that needed to be examined for the cluster analysis.

### Patient and public involvement

The study participants were not involved in the development of this study. The results of the study were not shared with the participants.

## Results

A total of 3,324 participants from all over Malaysia participated in this study. The mean age for participants was 43.0 ± 16.2, with a nearly equal male to female ratio. Majority of the participants were from West Malaysia (71.9%). Malay ethnic group accounted for 64.0% of the population, followed by Chinese (9.8%), Indigenous Sarawak (8.9%), Indigenous Sabah (7.8%), Indian (5.3%), and others (4.2%).

All ten head-and-face anthropometric dimensions measured in males were larger than in females, with a significant difference (*p* < 0.005) in sizes (Table 2). The findings also revealed that all head-and-face anthropometric dimensions differed significantly across birthplaces and ethnicity. Effect size measurement using Cohen's d showed that the effect is large for all the dimensions between sex (Cohen's d ≥ 0.8).

**Table 2:** Weighted head and facial dimensions according to sex, birthplace and race.

**Dimensions**	**Male (n = 1,556)**	**Female (n = 1,768)**	***P-*value**	**Cohen's d**
	**Mean ±SD**	**Mean ±SD**		
Bizygomatic breadth	141.5 ± 1.1	135.1 ± 0.9	<0.001	6.4
Minimum frontal breadth	100.8 ± 0.6	96.7 ± 0.5	<0.001	7.4
Bigonial breadth	111.5 ± 1.1	104.9 ± 1.0	<0.001	6.3
Menton-sellion length	122.2 ± 0.3	113.6 ± 0.3	<0.001	28.7
Interpupillary distance	64.9 ± 0.2	62.2 ± 0.1	<0.001	17.1
Head breadth	156.5 ± 1.0	152.5 ± 0.8	<0.001	4.4
Nose protrusion	18.0 ± 0.1	16.6 ± 0.1	<0.001	14.7
Nose breadth	43.2 ± 0.2	40.3 ± 0.1	<0.001	18.1
Nasal root breadth	17.8 ± 0.1	17.3 ± 0.1	<0.001	5.0
Subnasal-sellion length	51.1 ± 0.1	47.9 ± 0.2	<0.001	20.3
**Dimensions**	**East Malaysia (n** = **938)**	**West Malaysia (n** = **2,386)**	***P*** **-value**	**Cohen's d**
	**Mean** ±**SD**	**Mean** ±**SD**		
Bizygomatic breadth	139.3 ± 1.8	138.0 ± 1.1	<0.001	0.8
Minimum frontal breadth	100.5 ± 0.7	98.3 ± 0.6	<0.001	3.3
Bigonial breadth	110.9 ± 0.8	107.7 ± 1.1	<0.001	3.3
Menton-sellion length	117.5 ± 0.5	118.0 ± 0.2	<0.001	1.3
Interpupillary distance	63.2 ± 0.3	63.7 ± 0.1	<0.001	2.2
Head breadth	157.1 ± 1.0	153.9 ± 1.0	<0.001	3.2
Nose protrusion	17.1 ± 0.1	17.4 ± 0.1	<0.001	2.9
Nose breadth	41.6 ± 0.2	41.6 ± 0.1	<0.001	0.1
Nasal root breadth	17.7 ± 0.1	17.5 ± 0.1	<0.001	2.0
Subnasal-sellion length	49.3 ± 0.3	49.6 ± 0.1	<0.001	1.3
**Dimensions**	**Malay**	**Non-Malay**	***P*** **-value**	**Cohen's d**
	**Mean** ±**SD**	**Mean** ±**SD**		
Bizygomatic breadth	138.4 ± 0.7	138.1 ± 1.9	<0.001	0.2
Minimum frontal breadth	97.8 ± 0.7	99.9 ± 0.7	<0.001	0.3
Bigonial breadth	108.3 ± 0.6	108.1 ± 1.9	<0.001	0.1
Menton-sellion length	118.0 ± 0.3	117.9 ± 0.3	<0.001	0.3
Interpupillary distance	63.7 ± 0.1	63.5 ± 0.2	<0.001	1.3
Head breadth	154.4 ± 0.5	154.5 ± 1.9	<0.001	0.1
Nose protrusion	17.1 ± 0.1	17.5 ± 0.2	<0.001	2.5
Nose breadth	41.8 ± 0.1	41.3 ± 0.1	<0.001	5.0
Nasal root breadth	17.1 ± 0.1	17.5 ± 0.1	<0.001	4.0
Subnasal-sellion length	49.4 ± 0.2	49.7 ± 0.2	<0.001	1.5

Table 3 showed a summary of anthropometric statistics for males and females based on Mean, SD, Minimum, Maximum, Skewness, Kurtosis and Percentiles P5, P50, and P95. It was worth noting that the mean and standard deviation has been weighted to represent the Malaysian population.

**Table 3:** Head and facial dimensions by sex according to Mean, Minimum, Maximum, Skewness, Kurtosis, Percentiles P5%, P50%, P95%.

**Dimensions**	**n**	Mean^*^	SD^*^	**Minimum**	**Maximum**	**Skewness**	**Kurtosis**	**Percentile 5%**	**Percentile 50%**	**Percentile 95%**
**Male**										
Bizygomatic breath	1,556	141.5	1.1	100.0	175.0	−0.6	0.5	120.0	142.0	156.0
Minimum frontal breadth	1,556	100.8	0.6	67.4	144.5	0.2	2.2	87.5	100.5	114.6
Bigonial breadth	1,556	111.5	1.1	71.0	162.0	0.4	1.4	96.0	111.0	130.0
Menton-sellion length	1,556	122.2	0.3	94.9	172.4	0.3	1.8	109.7	121.9	134.7
Interpupillary distance	1,556	64.9	0.2	51.1	96.3	1.0	4.9	59.2	64.6	71.4
Head breadth	1,556	156.5	1.0	107.0	188.1	−0.7	2.3	141.0	157.0	170.0
Nose protrusion	1,556	18.04	0.1	11.6	26.8	0.5	0.8	15.0	17.9	21.7
Nose breadth	1,556	43.20	0.2	32.6	66.8	0.8	2.8	37.6	43.2	49.0
Nasal root breadth	1,556	17.8	0.1	11.1	26.2	0.5	0.7	14.8	17.7	21.2
Subnasal-sellion length	1,556	51.1	0.1	37.5	73.8	0.3	0.8	44.4	50.9	58.1
**Female**										
Bizygomatic breath	1,768	135.1	0.9	70.0	164.8	−0.9	1.8	115.0	136.0	150.0
Minimum frontal breadth	1,768	96.7	0.5	58.4	129.5	−0.4	1.3	83.4	96.7	109.0
Bigonial breadth	1,768	104.9	1.0	59.0	160.0	−0.1	2.6	90.0	105.0	120.0
Menton-sellion length	1,768	113.6	0.3	75.3	140.4	0.2	0.9	101.9	113.2	125.0
Interpupillary distance	1,768	62.2	0.1	52.2	77.4	0.5	0.8	56.7	62.0	68.1
Head breadth	1,768	152.5	0.8	109.0	218.6	−0.7	4.8	137.0	154.0	165.0
Nose protrusion	1,768	16.57	0.1	10.6	24.9	0.3	0.7	14.0	16.5	19.5
Nose breadth	1,768	40.34	0.1	29.7	56.8	0.5	0.9	35.3	40.2	46.1
Nasal root breadth	1,768	17.3	0.1	11.5	26.9	0.7	1.4	14.5	17.2	20.8
Subnasal-sellion length	1,768	47.9	0.2	33.2	65.7	0.1	0.3	41.3	47.8	54.8

* Weighted to represent population in Malaysia.

Head and facial dimensions have been found to be affected by various factors including sex, ethnicity, birthplaces, age, and BMI (Table 4). Sex was found to be a significant predictor for all the ten head and facial dimensions, with *p* < 0.001. These predictors can explain 3–29 percent of the variation (adjusted R^2^) in the desired outcome, with the most variation seen for menton-sellion length.

**Table 4:** The regression coefficients of regression analysis for anthropometric measurements by sex, ethnicity, birthplace, age, and BMI.

**Dimensions**	**Constant**	**Sex**	***P* value**	**Ethnicity**	***P* value**	**Birthplace**	***P* value**	**Age**	***P* value**	**BMI**	***P* value**	**Adjusted R^2^ (%)**	***P* value**
Bizygomatic breath	129.16	−6.70	<0.001	−0.79	0.072	1.25	0.007	−0.07	<0.001	0.57	<0.001	17	<0.001
Minimum frontal breadth	94.87	−4.58	<0.001	2.41	<0.001	0.65	0.085	–.07	<0.001	0.29	<0.001	13	<0.001
Bigonial breadth	95.20	−7.26	<0.001	−1.21	0.003	3.58	<0.001	−0.01	0.620	0.63	0.028	23	<0.001
Menton-sellion length	114.85	−8.96	<0.001	−1.28	<0.001	0.36	0.290	0.05	<0.001	0.21	<0.001	29	<0.001
Interpupillary distance	63.0	−2.87	<0.001	0.15	0.332	−0.63	<0.001	−0.03	<0.001	0.13	<0.001	17	<0.001
Head breadth	150.01	−4.12	<0.001	−0.50	0.170	3.07	<0.001	−0.64	<0.001	0.33	<0.001	11	<0.001
Nose protrusion	16.52	−1.48	<0.001	0.29	<0.001	−0.33	<0.001	0.03	<0.001	0.02	0.005	12	<0.001
Nose breadth	37.39	−3.02	<0.001	−5.42	<0.001	0.38	0.13	0.05	<0.001	0.15	<0.001	24	<0.001
Nasal root breadth	16.34	−0.47	<0.001	0.02	0.818	0.18	0.045	0.01	0.254	0.05	<0.001	3	<0.001
Subnasal-sellion length	49.88	0.07	<0.001	−0.58	0.001	0.04	0.852	0.07	<0.001	−0.06	<0.001	19	<0.001

Constant represents the regression intercept. The regression coefficients for categorical variables represent differences of male vs. female (reference), Malay vs. non-Malay (reference), East vs. West Malaysia (reference). Age and BMI are continuous variables.

In Table 5, we compared our findings among males and females to two previous studies with large sample sizes (China and NIOSH respiratory participants from the United States). The male participants in our study had smaller bizygomatic breadth, minimal frontal breath, bigonial breadth, and nose protrusion than the male participants in the comparison studies. Only two facial dimensions, interpupillary distance and nose breadth, were greater than those of the Chinese and NIOSH respirator studies. The menton-sellion and subnasal-sellion lengths were found to be shorter in the Chinese study but longer in the NIOSH study, whereas head breath was found to be larger in the Chinese study but smaller in the NIOSH study. The Chinese study had a wider nasal root breadth than this study, whereas this data was not available for the NIOSH study.

**Table 5:** Comparison of head and facial anthropometric measurement by sex in the current study, the NIOSH Respirator study, and the Chinese study.

**Dimensions**	**Present study**			**NIOSH Respirator study** **(****24****)**			**Chinese study** **(****5****)**		
	**n**	**Mean**	**±SD**	**n**	**Mean**	**±SD**	**n**	**Mean**	**±SD**
**Male**
Bizygomatic breath	1,556	141.5	1.1	2,543	143.5	6.9	2,026	147.5	4.7
Minimum frontal breadth	1,556	100.8	0.6	2,543	105.5	5.7	2,026	108.7	5.1
Bigonial breadth	1,556	111.5	1.1	2,543	120.4	10.4	2,026	119.0	8.5
Menton-sellion length	1,556	122.2	0.3	2,543	122.7	7.0	2,026	117.3	5.6
Interpupillary distance	1,556	64.9	0.2	2,543	64.5	3.6	2,026	64.2	2.7
Head breadth	1,556	156.5	1.0	2,543	153.0	6.0	2,026	157.2	5.3
Nose protrusion	1,556	18.0	0.1	2,543	21.1	2.7	2,026	18.9	1.9
Nose breadth	1,556	43.2	0.2	2,543	36.6	4.1	2,026	39.2	2.4
Nasal root breadth	1,556	17.8	0.1	2,543	No data	No data	2,026	18.3	1.9
Subnasal-sellion length	1,556	51.1	0.1	2,543	52.0	4.1	2,026	50.7	2.9
**Female**
Bizygomatic breath	1,768	135.1	0.9	1,454	135.1	6.5	974	139.9	6.3
Minimum frontal breadth	1,768	96.7	0.5	1,454	102.9	5.4	974	106.6	7.5
Bigonial breadth	1,768	104.9	1.0	1,454	110.1	8.9	974	114.2	10.6
Menton-sellion length	1,768	113.6	0.3	1,454	113.4	6.1	974	110.3	7.2
Interpupillary distance	1,768	62.2	0.1	1,454	61.9	3.5	974	61.0	3.5
Head breadth	1,768	152.5	0.8	1,454	146.8	5.6	974	150.5	7.1
Nose protrusion	1,768	16.6	0.1	1,454	19.8	2.7	974	17.7	2.4
Nose breadth	1,768	40.3	0.1	1,454	33.2	3.9	974	36.1	3.1
Nasal root breadth	1,768	17.3	0.1	1,454	No data	No data	974	17.3	2.2
Subnasal-sellion length	1,768	47.9	0.2	1,454	48.2	3.8	974	47.3	3.9

NIOSH Respirator study (24); Chinese study (5).

Four of the head and facial dimensions (interpupillary distance, head breadth, nose breadth and menton sellion) in our female participants were found to be the largest when compared to participants in the NIOSH respirator and Chinese study (Table 5). The bizygomatic breadth was of similar length as the NIOSH respirator study, but shorter than the Chinese study. Both our study and the Chinese study had the same nasal root length, but no comparison could be made for the NIOSH study. Our study had a narrower frontal breadth, a narrower bigonial breadth, and a shorter nose protrusion than the other two studies.

The participants were divided into ten groups using cluster analysis based on their homogeneity (Supplementary Table 1). From the MANOVA, Wilk's Lamda of ten groups yielded the highest result. The first group contained 0.99% of the samples, the second group contained 0.5%, and the remaining eight groups contained 0.4%.

## Discussion

There were significant differences in means of the head and facial anthropometric dimensions between sex, ethnicity and birthplace. A large sample size of more than 3,000 participants may have contributed to the statistical significance across groups for this study. In contrast, effect size is independent of the sample size (23). Thus, effect size was examined in the current study. The findings of current study showed that the differences between sex had a large effect size (≥0.8) across all 10 dimensions. Our findings are in accordance with previous local and international studies that showed statistically and practically significant differences in dimensions between sex (5, 14, 24, 25). It is worth noting that we are the only study which examined effect size in comparison to previous studies, thus comparison cannot be made.

Further analysis using Linear model showed that sex, ethnicity, birthplace, age and BMI were predictors for head and facial dimensions. Analysis using backward deletion model showed that sex (independent variable) was a significant predictor for all the 10 head and facial dimensions, followed by birthplace (independent variable), which affected all dimensions except subnasale-sellion length and BMI (independent variable), which influenced all dimensions except minimal frontal breadth.

When compared to participants from the United States (3) and China (5), our study population had a wider interpupillary distance and nose breadth for both male and female participants, but smaller bigonial breadth and minimal frontal breadth. However, we were not able to determine the statistical difference, as we did not have individual data of previous studies. Differences in the head and facial dimensions between these studies may have been influenced by genetic (26), environmental factors (27) and even the interaction of these two factors (28). Genetic factors have been proposed to play a significant role in the variation of head and facial dimensions (26). Whilst, environmental factor such as extreme temperature and humidity in the winter have been found to be associated with mid-facial morphology (27).

We acknowledge that the current study had some limitations. Head and facial anthropometry was not measured using the 3D stereophotogrammetry, but it should be noted that direct measurement and 2D photogrammetry were both validated tools for measuring head and facial anthropometry, and they are more feasible for a nationwide population study, particularly when conducted during the COVID-19 pandemic in 2020 to reduce contact time with participants. Another limitation is the lack of occupational information, which has been shown to be a significant predictor of head and facial dimensions (3). Our study population differed from previous study in that it was conducted among communities rather than across workplace sectors (3). Notably, while respirators are typically employed to protect workers against chemical and biological hazards in the workplace, respirators are particularly crucial during pandemics caused by airborne transmission, such as SARS-CoV-2. Furthermore, respirators are usually designed based on head and facial dimensions rather than occupations. Finally, the height was measured with a tape measure rather than a stadiometer. The main issue is that because our study was conducted in a community context, the rod could not be installed in certain locations due to uneven flooring. To minimize measurement bias, we used measurement tape that was specifically printed with stickers and taped to the walls and tent poles.

The main strength of current study is that it is the first large nationwide study on head and facial anthropometry of different ethnic groups in Malaysia with over 3,000 participants. Our total number of participants (3,324) was higher than that of the Chinese study (3,000) (5), but lower than that of the NIOSH study (3,997) (3). In comparison to the previous two large nationwide studies, our participants have almost equal number of male and female. Other strength of this study is the combination of direct and 2D measurement methods. This also indicate the important role that 2D photogrammetry, which has been shown to have high validity and reliability, can play in assessing specific head and facial morphologies in countries with limited 3D scanner resources. Another highlight of this study was the use of complex sample for analysis. It is essential in a multi-stage stratified sampling because it increases the precision of sample and ensures a representative sample from key groups (29).

Despite the fact that we have a multi-ethnic population, the cluster analysis revealed that our samples were very homogeneous, with more than 0.99% of our samples classified into the same group. This is surprising given that we have observed significant differences in the majority of head and facial dimensions across ethnicities and birthplaces. This data, however, indicated that, despite our different ethnicities, our genetics (28) affecting head and facial dimensions between ethnicities are likely to be comparable. The significance of this study was that the findings enabled us to uncover differences in dimensions between populations. We could use this information to consider the need of developing our own local respirators against biological and chemical hazards. We believe that our findings could assist in the design and sizing of respirators for Malaysians and possibly other Southeast Asian population because we share certain similarities.

## Data availability statement

The datasets used and/or analysed during the current study are available from the corresponding author on reasonable request.

## Ethics statement

Ethical approval was obtained from Medical Research and Ethics Committee (NMRR-20-1217-55489). Informed consent was obtained from all individual participants included in the study. The patients/participants provided their written informed consent to participate in this study.

## Author contributions

The study conception was by YCL, RS, and GTW. YCL and RS designed the study. YCL, ASAS, NM, MAP, RI, ZLC, and MHAM collected the data. YCL, ASAS, NM, MAP, MAO, MD, and RS conducted the statistical analysis and interpreted the results. YCL, ASAS, NM, and RS drafted the manuscript. All authors have read and approved the final version of the submitted manuscript.

## Funding

Funding for this study and publication were by the Ministry of Health, Malaysia.

## Conflict of interest

The authors declare that the research was conducted in the absence of any commercial or financial relationships that could be construed as a potential conflict of interest.

## Publisher's note

All claims expressed in this article are solely those of the authors and do not necessarily represent those of their affiliated organizations, or those of the publisher, the editors and the reviewers. Any product that may be evaluated in this article, or claim that may be made by its manufacturer, is not guaranteed or endorsed by the publisher.
