# Supplementary material for: Head and face anthropometric study for respirators in the multi-ethnic Asian population of Malaysia
Source: Front Public Health. 2022 Aug 26;10:972249. doi: 10.3389/fpubh.2022.972249 (PMC9459016; doi:10.3389/fpubh.2022.972249)
Supplement: Supplementary file 1 [file Table_1.DOCX]

**Supplemental Table 1: Cluster analysis to group participants based on multivariate similarities across samples**

|  | **Frequency** | **Percent** | **Valid Percent** | **Cumulative percent** |
| --- | --- | --- | --- | --- |
| 1 | 3295 | 99.1 | 99.1 | 99.1 |
| 2 | 15 | .5 | .5 | 99.6 |
| 3 | 2 | .1 | .1 | 99.6 |
| 4 | 1 | .0 | .0 | 99.7 |
| 5 | 1 | .0 | .0 | 99.7 |
| 6 | 3 | .1 | .1 | 99.8 |
| 7 | 1 | .0 | .0 | 99.8 |
| 8 | 4 | .1 | .1 | 99.9 |
| 9 | 1 | .0 | .0 | 100.0 |
| 10 | 1 | .0 | .0 | 100.0 |
| Total | 3324 | 100.0 | 100.0 |  |
